# Female patients at increased risk for adverse outcomes after acute limb ischemia

**DOI:** 10.1016/j.jvs.2025.08.026

**Published:** 2025-08-26

**Authors:** Mikayla N. Lowenkamp, Marissa C. Jarosinski, Kevin Li, Elizabeth A. Andraska, Ulka Sachdev-Ost, Rabih Chaer, Natalie D. Sridharan

**Affiliations:** a Division of Vascular Surgery, University of Pittsburgh Medical Center; b University of Pittsburgh School of Medicine.

**Keywords:** Acute limb ischemia, Sex disparities, Females, Adverse outcomes, ALI

## Abstract

**Objective:**

The under-representation of female patients in key trials results in a lack of sex-based guidelines regarding appropriate evaluation, diagnosis, and management of the female vascular patient. As a result, recent literature has found a difference in the amputation and mortality rates in female patients after treatment for acute limb ischemia (ALI). However, the reasons for outcome variability are unknown. The objectives of this study were to identify sex specific predictors of major amputation and mortality after intervention for ALI and sex-specific differences in the presentation, management, and outcome of patients who undergo revascularization for ALI.

**Methods:**

We included all adults who underwent revascularization for ALI at a multihospital health care system (2016–2023), excluding those who presented secondary to trauma, dissection, iatrogenic injury, popliteal aneurysms, or COVID. The terms male and female were used to delineate patient’s sex assignment at birth, were obtained from electronic health records, and were assumed to be congruent with gender references. Survival and amputation were evaluated using Kaplan-Meier and multivariable Cox regression with a priori and empirically selected covariates. Comprehensive subgroup analyses were conducted to assess risk of mortality and amputation.

**Results:**

We identified 548 patients, of whom 252 (46%) were female. Male patients were younger (64.4 ± 11.5 years vs 67.0 ± 15.3 years; *P* = .023), more likely to have coronary artery disease (*P* = .014), a smoking history (*P* < .001), and a history of prior revascularization (*P* < .001). Female patients were more likely to be hypercoagulable (*P* = .001) and less likely to present with acute-on-chronic disease (*P* < .001). Female patients were less frequently on a preoperative statin (*P* < .001) or antiplatelet agent (*P* = .004). Although there was no sex-based difference in Rutherford ALI classification upon presentation, female patients were more likely to go to the operating room within 24 hours (*P* = .024). There were no differences in the initial surgical approach (endovascular vs open). Female patients had an increased rate of death on univariable (*P* = .009) and multivariable (adjusted hazard ratio, 1.6; 95% confidence interval, 1.07–2.33) analyses. On subgroup analyses, female patients who were optimized medically on presentation achieved mortality rates similar to male patients. Although there was no difference in overall amputation rates, female patients who underwent an endovascular first approach were twice as likely to undergo amputation in comparison with males (odds ratio, 2.6; *P*_interaction_ = .01).

**Conclusions:**

Female patients who presented with ALI had higher mortality after revascularization, except for those who were medically optimized. They also had notably higher amputation rates after endovascular intervention. Further exploration of these disparities may allow for tailored intervention strategies by sex.

Acute limb ischemia (ALI) is a vascular catastrophe that is associated with high rates of morbidity and mortality, despite modern technological advancements.^1^ Recent epidemiological studies have demonstrated that, despite decreasing incidence and in-hospital amputation rates, mortality rates after an episode of ALI remain unchanged.^2^ Despite a decreasing incidence in ALI-related hospitalization in comparison with male patients, female patients continue to display an increased risk of in-hospital mortality.^2^

Disparate outcomes between the sexes (as assigned at birth) are detected across many vascular surgical conditions, and correlate with the under-representation of female patients in key studies and trials.^2–4^ This results in a lack of sex-based guidelines regarding the appropriate evaluation, diagnosis, and management of the female vascular patient.^3,4^

Recent literature has found a difference in the amputation and mortality rates in females, which, for the purposes of this report, is assumed to correlate with female sex at birth, after treatment for ALI.^5^ Prior studies however, have limited granularity and thus the reasons for outcome variability are unknown. Thus, the aims of this study were to identify sex-specific differences in presentation, management, and outcome, and to identify sex-specific predictors of major amputation and mortality after intervention for ALI. We hypothesized that, compared with those assigned male at birth, females presenting with ALI would exhibit worse limb salvage and mortality rates.

## METHODS

This retrospective cohort study was undertaken at a multihospital health care system and included all patients who underwent a revascularization procedure for ALI. This study used deidentified data in a retrospective manner and thus was deemed exempt by the University of Pittsburgh Institutional Review Board (STUDY23090078). The STROBE reporting guidelines were followed for this study.^6^

### Data source and patient cohort.

Patients were identified via review of the electronic health record (EHR) through Medical Archival Systems using Boolean searching, allowing for a comprehensive identification and review of patients who underwent revascularization for ALI at a multihospital health care system. Diagnosis was confirmed using manual chart review. We included those with less than 2 weeks of leg pain, with or without motor or sensory deficits, and with evidence of acute arterial occlusion who underwent any revascularization procedure. Patients who presented with ALI secondary to trauma, dissection, iatrogenic injury, popliteal aneurysm, or COVID-19 were excluded.

Patients were dichotomized based on sex assigned at birth and termed either male or female patient, which was abstracted from the EHR. Our EHR has only recently started capturing data on gender and were not consistently available.

### Outcomes.

The primary outcomes of interest were mortality and amputation. Primary end points were assessed longitudinally over a 5-year period after the index revascularization. These data were extracted from the her, which is integrated with other regional health systems to allow for more complete capture of the outcomes. Secondary outcomes included in-hospital complications, which included in-hospital cardiac or respiratory complications, and in-hospital mortality, which was extracted from chart review of the EHR. Cardiac complications were defined as any acute coronary syndrome (myocardial infarction or unstable angina) or heart failure exacerbation (documented echocardiographic evidence of reduced ejection fracture or diastolic dysfunction with symptoms of volume overload requiring diuresis. Respiratory complications were defined as respiratory failure requiring reintubation or new continuous positive airway pressure/bilevel positive airways pressure, pneumonia, or persistent oxygen requirements at discharge.

### Variables of interest.

Optimal medical therapy (OMT) was defined as nonsmoking patients on either an antiplatelet agent or anticoagulation and a statin.^7^ The etiology of the presentation was determined to be either embolic or acute on chronic in situ thrombosis. Acute-on-chronic disease was defined in patients with a prior intervention or in situ thrombosis. An embolic etiology was determined based on a history of atrial fibrillation, evidence of an embolizing lesion, or no history or evidence of atherosclerotic disease. Computed tomography angiography obtained before intervention, as well as intraoperative reports, were reviewed. Those with no evidence of vessel calcification on imaging and no mention of atherosclerotic burden in the operative report were deemed to have no evidence of atherosclerotic disease. Intervention modality was assigned as either open or endovascular and determined by the initial operative approach. Patients who had an endovascular attempt with subsequent conversion to open were counted as an endovascular approach with conversion. Multilevel disease was defined as occlusion of at least two arterial levels on presentation.

### Statistical analyses.

Continuous variables were presented as mean ± standard deviation or median (interquartile range), and categorical variables were presented as frequencies (%). Comparisons were made using either the Student *t* test or Mann-Whitney *U* test where appropriate. Kaplan-Meier survival analyses determined differences in mortality and amputation rates between the sexes. Cox proportional hazards modeling identified predictors of our primary outcomes of interest, namely, mortality and amputation. Variables were selected a priori based on clinical relevance and variables with significant between-group differences. These included sex, age, diabetes, coronary artery disease, hypercoagulable state, cancer history, smoking history, statin use, antiplatelet use, Rutherford classification, disease level, intervention modality, and time to the operating room. Subgroup analyses assessed the association between sex and mortality, or amputation stratified by age, race, hypercoagulable states, OMT, etiology, Rutherford classification, and intervention mortality. We estimated adjusted hazard ratios (HRs) for each subgroup using multivariable Cox regression models. To formally test for differences among subgroups, we included an interaction term between sex and the subgroup of interest and conducted a Wald test for interaction.

Analyses were performed using StataMP version 18.0 (Stata Corp). All tests were two sided, and a significance level of *P* ≤ .05 was considered statistically significant.

### Exploratory analysis.

After the identification of a subset of female patients who required amputation after endovascular intervention, we conducted a post hoc chart review of these patients to identify contributing factors and elucidate patterns. This analysis was descriptive in nature, and no formal statistical testing was performed.

## RESULTS

Five hundred forty-eight patients who presented with ALI and underwent a revascularization procedure between 2016 and 2023 met inclusion criteria: the average patient age was 65.6 ± 13.4 years, the majority were White (85.6%), and 252 (46%) were female. In terms of presentation, 259 (47.3%) presented with an embolic etiology vs an acute-on-chronic presentation. Most patients presented with Rutherford classification 2a or 2b (1, 20.8%; 2a, 41.1%; 2b, 33.4%; 3, 4.7%). The majority underwent an open intervention (n = 371 [68.1%]) with an overall 30-day amputation rate of 3% and a 30-day mortality rate of 9.7%. The median follow up time was 426 days (interquartile range, 87–928 days).

### Presentation.

Baseline demographics can be found in Table I. The female patients were older (67.0 ± 15.3 years vs 64.4 ± 11.5 years; *P* = .023), with no differences in self-reported race between cohorts (*P* = .29). Female patients were less likely to have a smoking history (156 (61.9%) vs 229 (77.4%; *P* < .001) and had fewer comorbid conditions, including preexisting coronary artery disease (76 [30.2%] vs 119 [40.2%]; *P* = .014) and prior coronary artery bypass grafting (15 [6.0%] vs 49 [16.6%]; *P* ≤ .001). Female patients were also less likely to have prior lower extremity stenting (39 [15.5%] vs 74 [25.0%]; *P* = .006) or bypass (44 [17.5%] vs 94 [31.8%]; *P* < .001). Female patients were more likely to carry a prior hypercoagulable diagnosis on presentation (31 [12.3%] vs 19 [6.4%]; *P* = .017). Despite carrying a greater rate of hypercoagulable disorders, there was no difference in preoperative anti-coagulation use (*P* = .69). However, female patients were less likely to be on an antiplatelet agent (48.8% vs 61.1%; *P* = .004) or a statin (41.3% vs 55.7%; *P* < .001) preoperatively. When restricting the analysis to patients with preexisting coronary artery disease or a prior peripheral artery disease (PAD) intervention, there were no significant differences in the overall OMT prescription rates (*P* = .17 and .078, respectively).

In terms of presentation (Table II), female patients trended toward earlier presentation in relation to symptom onset, although this difference was not statistically significant (*P* = .063). However, female patients were more likely to go to the operating room within 24 hours of symptom onset (82.9% vs 75.0%; *P* = .024). There were no differences in Rutherford classification on presentation. Female patients were less likely to present with acute-on-chronic limb ischemia (41.3% vs 62.5%; *P* < .001) and, conversely, more likely to present with an embolic etiology. Of the 58.7% of females who presented with an embolic ALI event, 21.5% had a cardiac source, 5.1% had a thoracic aortic source, and 14.7% had an abdominal aorta or iliac source. Males were less likely to have an embolic lesion (37.5%) the distribution is as follows: 12.8% with cardiac source, 3.0% with thoracic aortic source, and 20.3% with abdominal aorta/iliac source. The remainder in each cohort were without a clear source. As far as anatomical distribution, female patients were less likely to have tibial involvement (46.0% vs 55.7%; *P* = .023). There was no difference in aortoiliac or femoropopliteal involvement between the groups. However, male patients were more likely to have multilevel occlusive involvement (*P* = .032).

### Treatment.

There were no differences in the initial revascularization strategy; equal proportions of males and females received endovascular (female, 29.9% vs male, 33.7%; *P* = .34) or open interventions (Table II). Endovascular interventions included thrombolysis or percutaneous thrombectomy. Similar proportions of patients received thrombolytic therapy (female patients, 19.8% vs male patients, 24.0%; *P* = .24) or a percutaneous intervention. There was no difference between the cohorts in patients who required an open conversion. In the open intervention subgroup (open thrombectomy or bypass), female patients were less likely to undergo a bypass (13.6% vs 20.7%; *P* = .03).

### Outcomes.

Despite greater in-hospital respiratory complication rates (11.9% vs 6.8%; *P* = .037), there were no differences between the groups in terms of cardiac events or in-hospital mortality. Female patients were less likely to be discharged on an antiplatelet agent (75% vs 85.5%; *P* = .002), with no differences in discharge anti-coagulation prescription rates (Table III).

**Fig 1.**
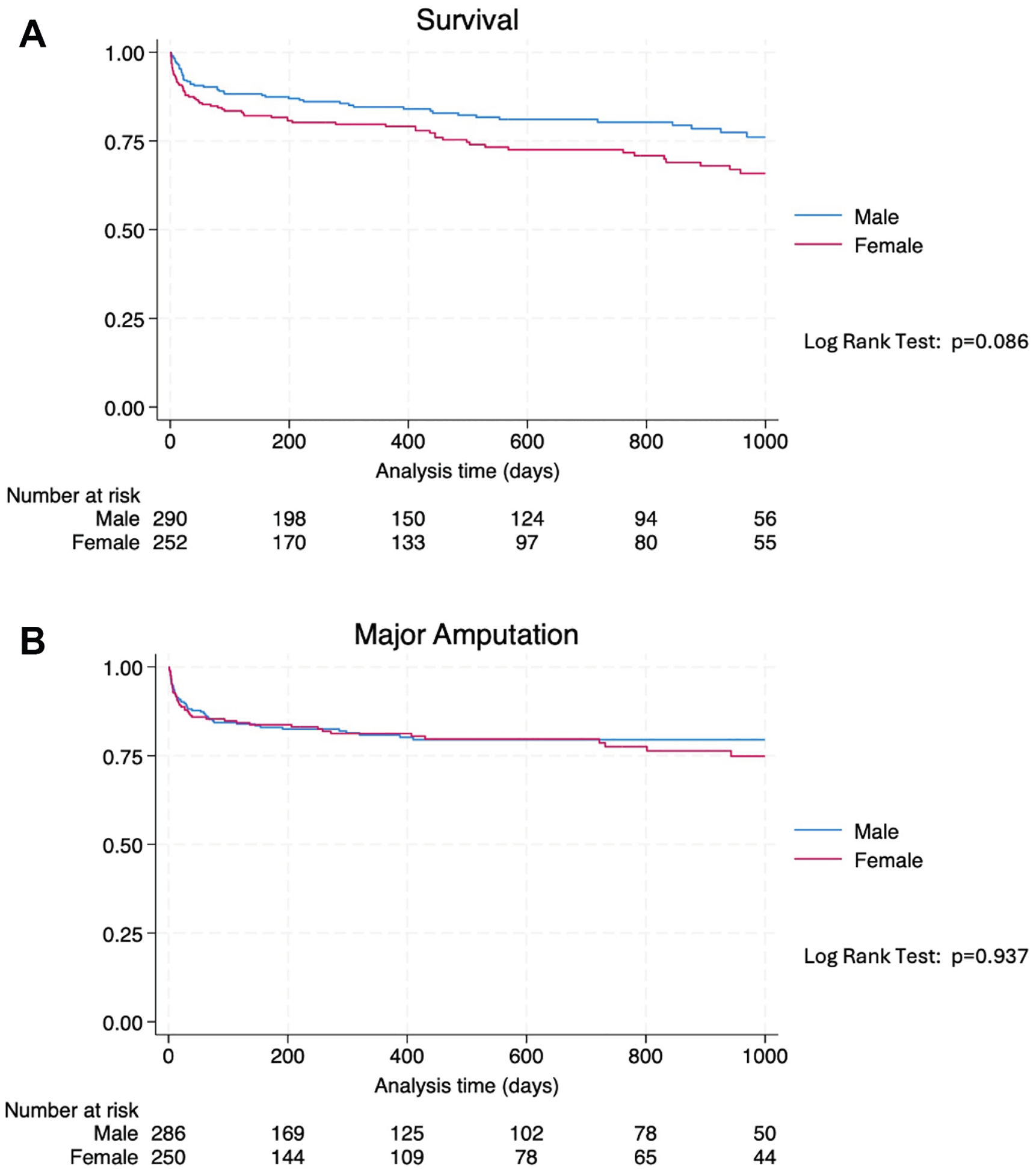
Kaplan-Meier time-to-event analysis of long-term mortality **(A)** and major amputation **(B)** by sex.

**Fig 2.**
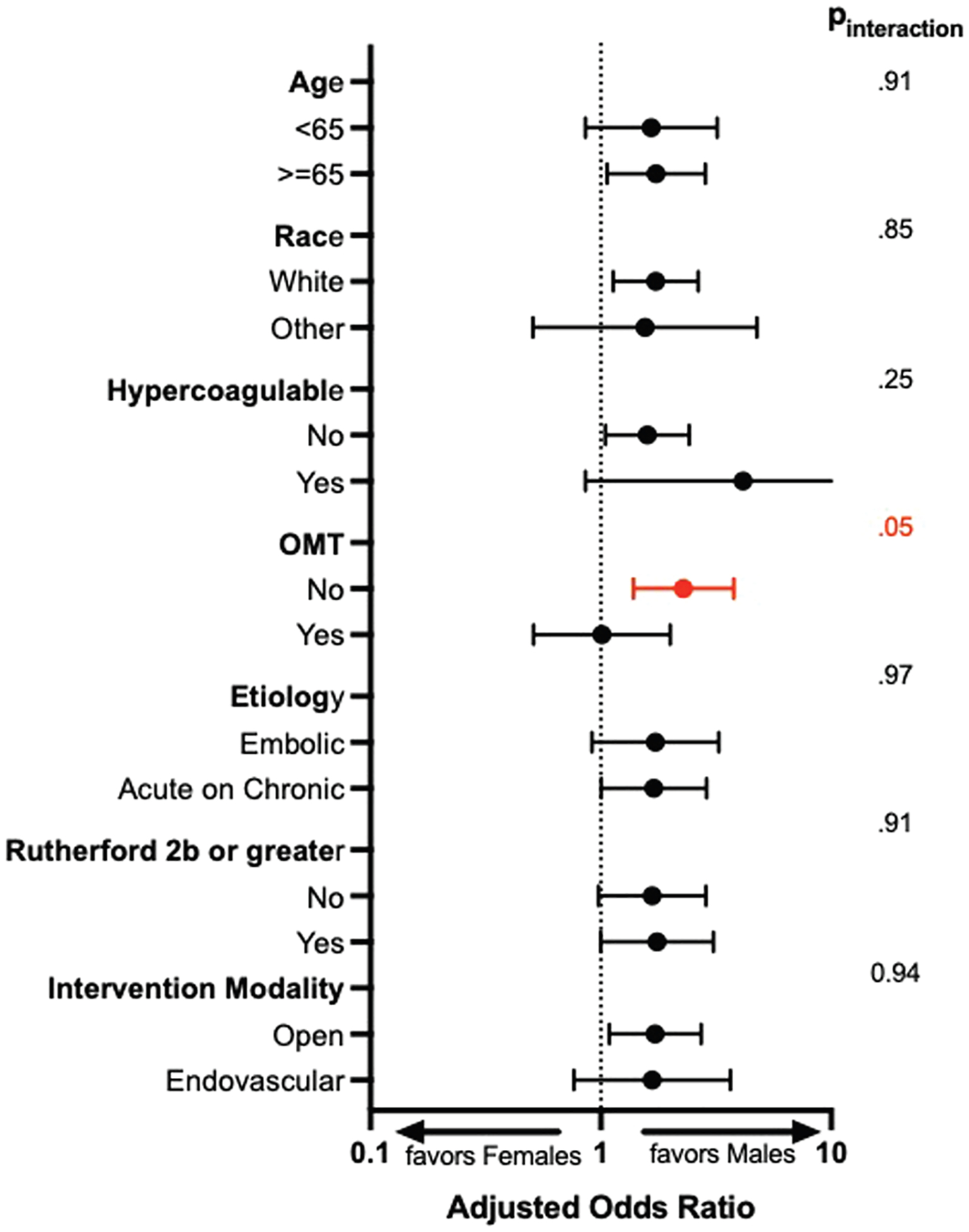
Mortality risk among subgroups. *OMT*, Optimal medical therapy.

On univariate analysis, female patients had lower long-term survival in comparison with male patients after lower extremity revascularization (*P* = .009) (Fig 1). On multivariable modeling, female patients had significantly increased risk of death (adjusted HR [aHR], 1.58; 95% confidence interval [CI], 1.07–2.33; *P* = .022) after adjusting for comorbidities, presentation and interventions (Supplementary Table I, online only). Advanced age, a cancer history, and advanced Rutherford classification were all predictors of mortality. The association between female sex and mortality was ubiquitously observed among the subgroups except for those medically optimized (Fig 2) (*P*_interaction_ = .05). Female patients who were not on medical therapy had more than twice the hazards of death in comparison to males not medially optimized (aHR, 2.29; 95% CI, 1.38–3.78). However, medically optimized female patients approached similar death risks as medically optimized males (Fig 2).

**Fig 3.**
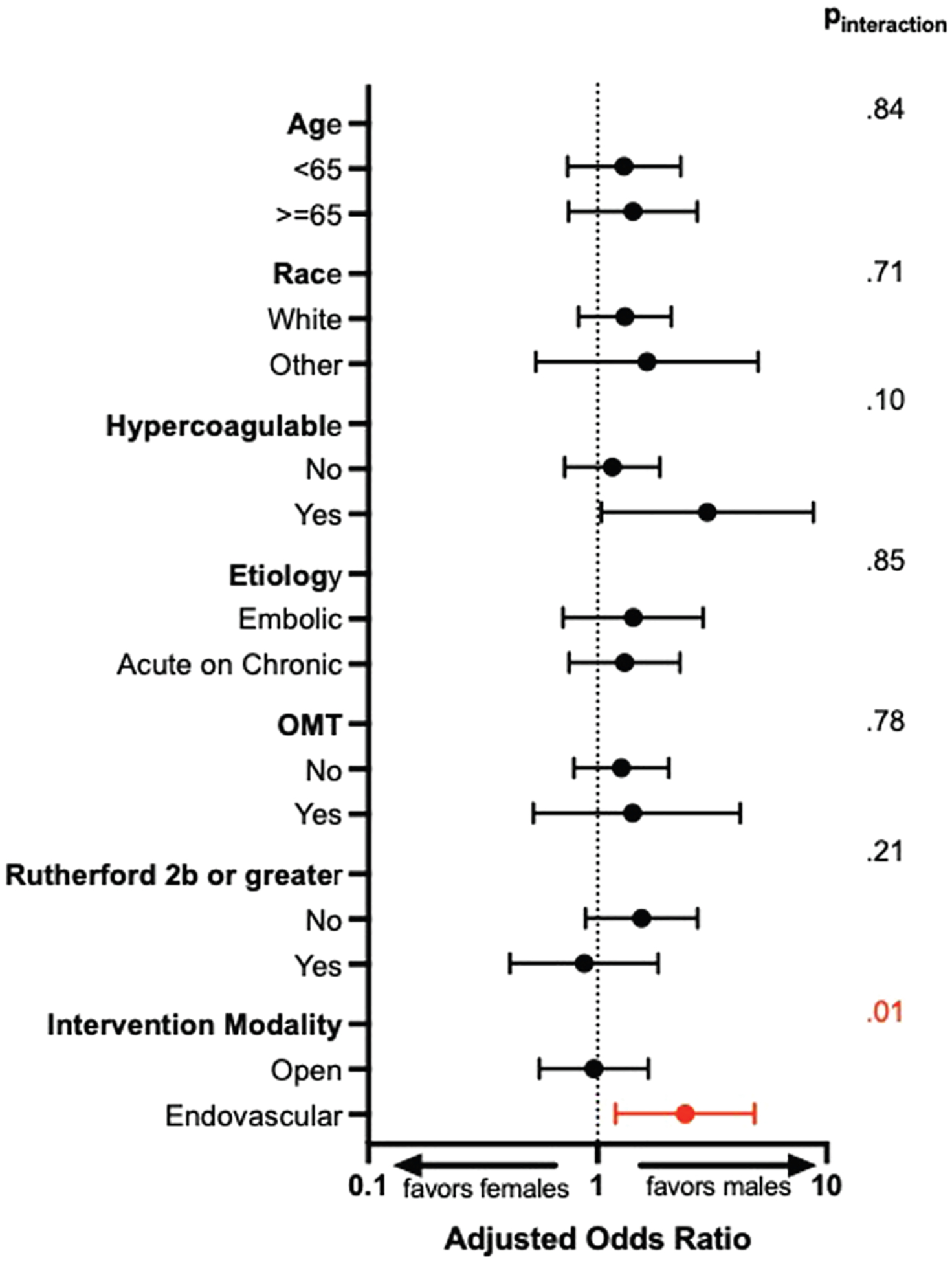
Amputation risk among subgroups. *OMT*, Optimal medical therapy.

There were no differences in major amputation rates between males and females on univariable analysis (Fig 1) (*P* = .937). On multivariable analysis, sex was not a significant predictor of amputation (Supplementary Table II, online only) (aHR, 1.26; 95% CI, 0.82–1.95; *P* = .291). However, advanced Rutherford classification, tibial involvement, and acute-on-chronic presentations were all associated with increased hazards of amputation (Supplementary Table II, online only). The association between sex and amputation was ubiquitously observed among subgroups, except for the intervention modality (Fig 3) (*P*_interaction_ = .04). Female patients who underwent an endovascular revascularization experienced more than twice the hazards of subsequent amputation in comparison to males who had an endovascular intervention (Fig 3) (aHR, 2.41; 95% CI, 1.99–4.86).

### Exploratory data analysis.

There were 21 female patients who underwent an endovascular intervention and subsequent amputation. The average age of this cohort was 63.7 ± 15.2 years and 18 (85.7%) were White. Eleven (52.4%) had a prior vascular intervention, 7 of which were prior endovascular interventions. The majority of this subgroup presented with tibial occlusions (n = 19 [90.5%]). In terms of modality chosen, 17 (81.0%) underwent overnight thrombolysis and 12 (57.1%) underwent percutaneous thrombectomy. Despite these attempts, in 16 cases (76.2%), tibial outflow was unable to be reestablished. Twelve patients (57.1%) underwent an in-hospital amputation; the remainder were completed at a subsequent hospitalization.

## DISCUSSION

The under-representation of female patients in clinical trials has resulted in a lack of sex-specific guidelines regarding the appropriate diagnosis, management, and operative intervention of the female vascular patient.^4,5,8^ Despite the equal incidence of ALI between sexes, female patients comprise only 30% of participants in ALI clinical trials.^9–11^ As a result, disparities in outcomes between males and females in vascular surgical and interventional procedures are increasingly evident.^3,5^ Additionally, the guidelines on ALI are sparse and fail to consider sex as a biologic variable.^12^ Using a granular dataset at a large-volume center, we explored ALI outcomes by sex.

Female patients were older than male patients, with fewer prior cardiovascular diagnoses or procedures. Female patients went to the operating room sooner than male patients despite presenting with a similar Rutherford classification. This may be because females were less likely to have acute-on-chronic disease, with less collateral reserve, and differing presenting symptoms that were not captured by the Rutherford classification. Both groups had similar intervention strategies. However, female patients had greater mortality. Female patients who were not on medical therapy fared the poorest. Medically optimized female patients exhibited similar mortality rates to male patients. Overall, females exhibited similar amputation rates to males, except in those who underwent an endovascular intervention. Females who underwent an endovascular intervention exhibited twice the amputation risk.

Although embolic and thrombotic etiologies may represent distinct processes with differing profiles, the outcomes these patients were analyzed together to preserve statistical power and reflect the heterogenous nature of ALI in clinical practice. The subgroup analyses that compared the differing etiologies demonstrated that sex-based differences in both mortality and amputation risk persisted across both embolic and acute-on-chronic presentations.

The disparate outcomes in mortality after intervention for ALI are well-documented.^2,5,13^ A large, propensity-matched cohort reported by Chihade et al^5^ evaluated ALI outcomes across males and females in an administrative insurance dataset. In their cohort, female patients were also older, with fewer cardiovascular comorbidities, likely representing a delay in diagnosis of PAD in this patient population per the authors. They believe these outcomes can be explained by a combination of advanced age, comorbid conditions, delayed presentation, hormonal factors, and smaller vessel diameter.

The greater mortality risk in females with ALI was again confirmed in an epidemiological study on ALI in a large National Inpatient Sample.^2^ Female patients were more likely to die despite controlling for age, demographics, and comorbidities. Despite the declining trends in ALI incidence and amputation overall, mortality in the female patient population has stagnated.^2^ The authors highlight the under-representation of female patients in large thrombolysis trials as an explanation for the lack of understanding of the factors that contribute to these disparate outcomes.

Prior work in other vascular domains has hypothesized that female patients present later in their disease state, which may account for the disparate outcomes.^3^ In our study, female patients were less likely to have acute-on-chronic ischemia, which may represent a delayed PAD diagnosis. However, our results also showed that female patients presented within a similar time frame after symptom onset with a similar Rutherford classification and went to the operating room sooner after presenting to the hospital in comparison with their male counterparts. Worse outcomes despite more expeditious care in the hospital indicates a potential disparity in care that lies before presentation to the hospital.

On subgroup analysis, female patients who were medically optimized had similar mortality rates compared with male counterparts. However, females not on OMT had more than twice the rate of mortality in comparison with males not on OMT. Although in our study, OMT rates were similar between sexes with preexisting PAD interventions, the literature consistently demonstrates that female patients have lower rates of receiving antiplatelet and statin therapy for PAD, which may portend poorer limb salvage and mortality benefits.^3,14–18^ These findings of lower rates of goal-directed medical therapy and subsequent worse outcomes in females have also been described in the coronary artery disease literature.^19–21^ This finding highlights a global need to develop strategies to minimize sex disparities in cardiovascular care. These cumulative findings present an actionable opportunity for vascular providers to address and potentially decrease treatment disparities in the female patient population.

Despite similar limb salvage rates overall, female patients who underwent endovascular treatment had higher amputation rates than their male counterparts on subgroup analysis. With the evolution of endovascular management over the past decade, the literature regarding the endovascular management of ALI is sparse. In the PAD literature, female patients undergoing endovascular intervention demonstrated more severe and complex lesions than their male counterparts, with overall higher rates of death, myocardial infarction, and amputation.^22^ The authors cautioned providers that procedural complications and prognosis may be worse in female patients undergoing endovascular therapy for PAD. Our study also elicits a potential concern over amputation risk in female patients who undergo an endovascular intervention. On exploratory analysis, we found that female patients with failed endovascular interventions had high rates of tibial involvement and in the majority of cases tibial outflow was unable to be reestablished. Ruiz et al^13^ looked at sex-based differences in outcomes after endovascular-first revascularization for ALI and showed lower amputation-free survival rates. The globally smaller vessel size in female patients could account for difference in limb salvage outcomes.^3^ Prior work on the endovascular management of femoropopliteal disease has demonstrated that small vessel diameter is associated with inferior outcomes.^23^ This factor may also apply to endovascular interventions for ALI. Regardless, it is crucial that future clinical trials studying endovascular devices recruit an equal number of female patients to reflect the equal incidence of ALI in female and male patients. This strategy will ensure appropriate application of endovascular therapies for female patients.

Many limitations of this study are inherent to its retrospective nature. Although the small cohort provided granular data regarding symptomology and timing that are not accounted for in other datasets, we are limited in further analysis, specifically regarding endovascular subgroups, given the smaller cohort. Additionally, endovascular techniques and PAD awareness have changed throughout the study timeline, which may have led to poorer outcomes documented in this study. This dataset also did not account for access to care, which may be highly variable and impact medication prescriptions. Last, cause of death data were not reliably available from the EHR and was unable to be accounted for.

In summary, there are major gaps in knowledge on the clinical presentation, diagnosis, and management of the female vascular patient with a resultant lack of sex-specific guidelines. We highlighted sex differences in those presenting with and undergoing treatment for ALI. Specifically, female patients presented with similar ALI severity, more embolic lesions, less multilevel disease, and still experienced greater mortality overall with more major amputations after endovascular interventions. This result may represent a distinct ALI phenotype. Ongoing research on this topic is essential to develop sex-specific diagnostic criteria and intervention recommendations to optimize care in the female patient population. This study also highlights the importance of medical optimization, specifically in the female patient population. The ALI encounter provides a meaningful and tangible opportunity to ensure all patients are medically optimized. In addition to ongoing research and improving medical management, vascular surgeons should strive to ensure heterogenous enrollment in future clinical trials to ensure all patients are well-represented in specialty advancements.

## CONCLUSIONS

There are notable disparities in the diagnosis, management, and outcomes of the female vascular patient. Female patients who present with ALI were less likely to be medically optimized at presentation, which suggests a potential underdiagnosis and management of vascular disease. They had higher rates of long-term mortality after revascularization except for those medically optimized. Female patients also had notably higher amputation rates following endovascular interventions. Further exploration of these disparities may allow for tailored intervention strategies by sex in those presenting with ALI.

## Supplementary Material

supp table 1

supp table 2

## Figures and Tables

**Table I.** Baseline demographics and past medical history by sex

Variable	Female (n = 252)	Male (n = 296)	*P* value
Demographics			
Age, years	67.0 ± 15.3	64.4 ± 11.5	.023
Race			.29
White	216 (85.7)	255 (86.1)	
Black	26 (10.3)	27 (9.1)	
Asian	0 (0.0)	4 (1.4)	
Other	10 (4.0)	10 (3.4)	
Past medical history			
Diabetes	85 (33.7)	78 (26.4)	.06
Hypertension	174 (69.0)	205 (69.3)	.96
Hyperlipidemia	99 (39.3)	133 (44.9)	.18
Coronary artery disease	76 (30.2)	119 (40.2)	.014
Hypercoagulable	31 (12.3)	19 (6.4)	.017
Smoking history	156 (61.9)	229 (77.4)	<.001
Prior coronary artery bypass	15 (6.0)	49 (16.6)	<.001
Prior lower extremity bypass	44 (17.5)	94 (31.8)	<.001
Prior lower extremity stent	39 (15.5)	74 (25.0)	.006
Preoperative medications			
Antiplatelet	123 (48.8)	181 (61.1)	.004
Statin	104 (41.3)	165 (55.7)	<.001
Anticoagulation	71 (28.2)	88 (29.7)	.69

Values are mean ± standard deviation or number (%).

**Table II.** Presentation and operative characteristics by sex

Variable	Female (n = 252)	Male (n = 296)	*P* value
Present within 24 hours of symptom onset	116 (46.0)	113 (38.2)	.063
Transfer from outside hospital	142 (56.3)	154 (52.0)	.31
OR within 24 hours	209 (82.9)	222 (75.0)	.024
Rutherford classification			
1	56 (22.2)	58 (19.6)	
2a	104 (41.3)	121 (40.9)	
2b	78 (31)	105 (35.5)	
3	14 (5.6)	12 (4.1)	.59
Acute-on-chronic limb ischemia	104 (41.3)	185 (62.5)	<.001
Multilevel occlusion	185 (73.4)	240 (81.1)	.032
Aortoiliac involvement	111 (44.0)	113 (38.2)	.16
Femoropopliteal involvement	228 (90.5)	275 (92.9)	.30
Tibial involvement	205 (69.3)	144 (57.1)	.003
Operative strategy			.34
Endovascular	75 (29.9)	99 (33.7)	
Open	176 (70.1)	195 (66.3)	
Thrombolysis	50 (19.8)	71 (24.0)	.24
Conversion to open	8 (10.7)	5 (5.1)	.16
Open thrombectomy	166 (65.9)	186 (63.1)	.49
Bypass	34 (13.6)	61 (20.7)	.03

*OR*, Operating room.

Values are number (%).

**Table III.** In-hospital complications and discharge medications

Variable	Female (n = 252)	Male (n = 296)	*P* value
Respiratory failure	30 (11.9)	20 (6.8)	.037
Cardiac failure	20 (7.9)	13 (4.4)	.082
Mortality	25 (9.9)	20 (6.8)	.19
Discharge anticoagulation	194 (77.0)	232 (78.4)	.70
Discharge antiplatelet	189 (75.0)	253 (85.5)	.002

Values are number (%).

## ARTICLE HIGHLIGHTS

- **Type of Research:** Single-center retrospective cohort study
- **Key Findings:** There were notable sex differences in 548 patients (46% female) who underwent intervention for acute limb ischemia. Females had a 58% increase in long-term mortality. Female patients who underwent an endovascular intervention had twice the odds of major amputation in comparison with males undergoing endovascular interventions.
- **Take Home Message:** Females who present with acute limb ischemia have greater mortality after intervention and greater risk of major amputation after endovascular intervention.
