# Supplementary material for: Female patients at increased risk for adverse outcomes after acute limb ischemia
Source: J Vasc Surg. Author manuscript; Available in PMC 2026 Apr 9. (PMC13065349; doi:10.1016/j.jvs.2025.08.026)
Supplement: supp table 1 [file NIHMS2161632-supplement-supp_table_1.pdf]

Supplementary Table I (online only) Cox proportional hazards modeling of mortality

Variable	aHR	95% CI		*P* value
Female sex	**1.58**	**1.07**	**2.33**	**.022**
Age	**1.04**	**1.02**	**1.06**	**.000**
Diabetes	1.21	0.81	1.79	.351
Coronary artery disease	1.48	1.00	2.19	.052
Hypercoagulable disease	0.59	0.31	1.11	.102
Cancer history	**1.67**	**1.06**	**2.63**	**.028**
Smoking history	1.38	0.88	2.16	.159
Antiplatelet agent	0.97	0.64	1.48	.903
Statin	1.25	0.84	1.85	.268
Rutherford classification (1 ref)				
2a	1.10	0.62	1.96	.739
2b	**1.85**	**1.02**	**3.37**	**.044**
3	**3.75**	**1.60**	**8.79**	**.002**
Aortoiliac involvement	0.79	0.53	1.19	.259
Tibial involvement	1.05	0.73	1.52	.792
Acute-on-chronic presentation	1.15	0.77	1.72	.503
Endovascular intervention	0.71	0.43	1.17	.175
Time to OR (<24 hours ref)				
6-24 hours	1.13	0.73	1.73	.586
>24 hours	0.59	0.34	1.02	.059

aHR, Adjusted hazard ratio; *CI*, confidence interval; *OR*, operating room.

Boldface entries indicate statistical significance.
