# Supplementary material for: Female patients at increased risk for adverse outcomes after acute limb ischemia
Source: J Vasc Surg. Author manuscript; Available in PMC 2026 Apr 9. (PMC13065349; doi:10.1016/j.jvs.2025.08.026)
Supplement: supp table 2 [file NIHMS2161632-supplement-supp_table_2.pdf]

Supplementary Table II (online only) Cox proportional hazard model of major amputation

Variable	aHR	95% CI		*P* value
Female sex	1.26	0.82	1.95	.291
Age	0.99	0.97	1.01	.389
Diabetes	1.37	0.88	2.14	.168
Coronary artery disease	0.85	0.53	1.35	.486
Hypercoagulable disease	1.06	0.62	1.81	.835
Cancer history	1.39	0.77	2.52	.28
Smoking history	0.76	0.47	1.24	.274
Antiplatelet agent	0.82	0.50	1.33	.413
Statin	1.07	0.67	1.70	.79
Rutherford classification (1 ref)				
2a	1.14	0.65	2.02	.65
2b	1.58	0.83	2.99	.163
3	**4.87**	**1.80**	**13.13**	**.002**
Aortoiliac involvement	0.74	0.46	1.19	.214
Tibial involvement	**3.44**	**2.14**	**5.52**	**<.001**
Acute-on-chronic presentation	**2.09**	**1.31**	**3.34**	**.002**
Endovascular intervention	1.06	0.65	1.73	.825
Time to OR (<24 hours ref)				
6-24 hours	1.08	0.65	1.81	.766
>24 hours	1.16	0.67	2.02	.592

***aHR*, Adjusted hazard ratio; *CI*, confidence interval; *OR*, operating room.**

Boldface entries indicate statistical significance.
